# Predictors of Semen Parameters Decline Following the Microsurgical Varicocelectomy

**DOI:** 10.7759/cureus.45061

**Published:** 2023-09-11

**Authors:** Daniel R Greenberg, Matthew T Hudnall, Bailey N Goyette, Richard J Fantus, Justin M Dubin, Robert E Brannigan, Joshua A Halpern

**Affiliations:** 1 Department of Urology, Northwestern University Feinberg School of Medicine, Chicago, USA; 2 Department of Urology, University of Missouri, Columbia, USA; 3 Department of Urology, University of Kansas, Kansas City, USA; 4 Department of Urology, Memorial Healthcare, Aventura, USA

**Keywords:** varicocelectomy, andrology and microsurgery, oligospermia, male factor infertility, semen analysis

## Abstract

Objective

Varicocele is considered the most common reversible cause of male infertility. However, some men do not clinically improve after surgical repair. We aimed to identify preoperative factors associated with decreased semen parameters and clinical “downgrading” of total motile sperm count (TMSC) following varicocelectomy.

Methods

We examined men with preoperative laboratory testing and pre- and postoperative semen analyses (SA) who underwent varicocelectomy between 2010 and 2020. Ejaculate volume, sperm motility, sperm concentration, TMSC, and clinical grade of TMSC (in vitro fertilization: <5M sperm, intrauterine insemination: 5-9M sperm, natural pregnancy: >9M sperm) were used to determine postoperative outcomes. Demographic and clinical factors were compared between cohorts.

Results

Among 101 men who underwent varicocelectomy, 35 (34.7%) had decreased postoperative TMSC with a median follow-up of 6.6 months (interquartile range 3.9-13.6 months). Eleven (10.9%) men experienced TMSC clinical “downgrading” following surgery. Clinical grade III varicocele was significantly associated with decreased sperm motility on postoperative SA (OR 4.1, 95% CI 1.7-10.0, p=0.002), and larger left testicle volume (OR 1.4, 95% CI 1.1-1.8, p=0.02) was associated with clinical “downgrading” after varicocelectomy.

Conclusion

A small but significant proportion of men experienced a “downgrading” of semen parameters after varicocelectomy. Larger left testis size was associated with clinical downgrading, whereas clinical grade III varicoceles were associated with lower post-treatment sperm motility. These data are critical for preoperative patient counseling.

## Introduction

Varicocele is a common medical condition affecting up to 15% of men [1]. Varicoceles are caused by valvular dysfunction within the pampiniform venous plexus, resulting in dilated or tortuous veins - often noted as a “bag of worms” on physical exam. Poor gonadal venous drainage increases scrotal temperature and results in progressive testicular atrophy and impaired spermatogenesis from scrotal hyperthermia and hypoxia [2,3]. Hypogonadism, testicular size and growth discrepancy, persistent pain, and infertility or subfertility can be noted clinically in a subset of affected men [4]. Previous studies show that varicoceles are present in 21-41% of men presenting with primary infertility, and over 70% of men with secondary infertility [5].

Surgical repair of clinical varicocele has been shown to increase sperm concentration, motility, and morphology in the majority of men [6,7]. It has also been proven to clinically “upgrade” couples’ eligibility for intrauterine insemination (IUI), and increase pregnancy rates, live birth rates, and sperm retrieval rates in couples undergoing assisted reproduction [8-10]. It is therefore considered the most common reversible cause of male infertility. Given this, current American Urological Association (AUA) and American Society of Reproductive Medicine (ASRM) guidelines recommend treatment of varicoceles for men attempting to conceive, who have palpable clinical varicocele, infertility, and abnormal semen parameters [11].

Despite its proven efficacy, a small portion of men experience no clinical benefit or even decreased fertility potential following varicocele repair. While previous studies have identified numerous factors associated with increased semen parameters following varicocelectomy including laterality, higher preoperative sperm concentration and total motile sperm count (TMSC), and lower baseline follicle stimulating hormone (FSH), there is a paucity of data focused on identifying factors associated with poor outcomes following varicocelectomy [12]. Given that varicocelectomy is an elective procedure intended to improve semen parameters and fertility but with the potential for the opposite outcome, it is critical that patients and providers understand the potential predictors of a negative clinical response. This may substantially impact clinical decision-making. We sought to identify prognostic factors associated with decreased semen parameters following varicocele repair. We hypothesize that specific patient clinical and demographic factors are associated with decreased semen parameters following varicocelectomy.

## Materials and methods

We retrospectively reviewed our institutional electronic database to identify adult men (18 years of age or older) who underwent either unilateral or bilateral microsurgical varicocelectomy between 2010 and 2020. We included all patients who underwent initial evaluation by a reproductive urologist (RU) at our institution, had preoperative laboratory testing, and both pre- and postoperative semen analyses (SA) available for review. We queried our database to obtain baseline patient demographic information including age at date of surgery, race, and ethnicity. Clinical variables included body mass index (BMI), testicular volume on clinical exam (compared to orchidometer), and preoperative laboratory values including serum FSH, luteinizing hormone (LH), and morning serum testosterone (T). Diagnosis of varicocele was made on clinical examination by an RU and classified in accordance with the Dubin grading system [13]. Diagnosis of varicocele by scrotal ultrasound was not required as inclusion criteria. Additionally, men with subclinical varicoceles detected only on ultrasound were excluded, as it is our clinical practice not to perform varicocelectomy on these men, in accordance with AUA/ASRM guidelines. This study was reviewed and exempted by the Institutional Review Board (IRB).

All varicocelectomies were performed by four fellowship-trained RUs via a microsurgical, sub-inguinal approach. This approach has been described extensively [14]. In brief, a 3 cm sub-inguinal incision was made inferior to the external inguinal ring, following which the spermatic cord was delivered. Using an operative microscope, the testicular artery or arteries were identified with the assistance of Doppler ultrasound and spared. All spermatic cord veins were then ligated using silk ties.

The primary outcomes of interest were changes in semen parameters and clinical “downgrading” on postoperative SA following surgical correction of varicocele. Preoperatively, men were categorized into three groups according to the type of assisted reproductive technology (ART) they would be eligible for and recommended given their baseline TMSC. Men with TMSC <5 million were considered candidates for in vitro fertilization (IVF), 5-9 million for IUI, and >9 million for natural pregnancy (NP) [10]. Postoperative SA was obtained at the last follow-up at least two months following the date of surgery. Ejaculate pH, ejaculate volume, sperm concentration, strict morphology, sperm motility, and TMSC were analyzed. Men were then recategorized after varicocele repair into the same three groups (IVF, IUI, NP) to determine changes in ART candidacy postoperatively. Downgrading was defined as a change to a lower TMSC category at follow-up, and upgrading was defined as a change to a higher TMSC category at follow-up. All semen processing and SA were performed in accordance with the 2010 WHO Laboratory Manual for the Examination and Processing of Human Semen (5th Edition) [15].

Data were analyzed using Stata v15.1 (StataCorp, 2020, College Station, TX) statistical software. Categorical variables are presented as n (%) and were analyzed using Fisher’s exact test or c2 as appropriate. The distribution of continuous data was evaluated by means of the Kolmogorov-Smirnov test. Continuous variables are presented as median (interquartile range) and were analyzed using the student’s t-test. Nonparametric data was presented as median (interquartile range) and were analyzed using the Mann-Whitney U (Wilcoxon rank sum) test. Univariate and multivariable logistic regression was used to determine the association of demographic and clinical factors associated with our primary outcome. All tests of significance were two-sided, and a p-value of <0.05 was deemed statistically significant.

## Results

Our initial retrospective database search captured 160 patients, 126 of which had pre- and postoperative SA available for review, and 101 who also had preoperative laboratory testing and therefore were included in the final analysis. Of the total 101 men included, 45 (44.6%) men had multiple postoperative SA. The median age at the time of surgical repair was 33 years old (IQR 30-36 years old). Varicocelectomy repair was bilateral in 55 (54.5%) patients and unilateral left-side in 46 (45.5%) of patients. There were no unilateral right-side varicocelectomies. Within the cohort, four patients (4.1%) had clinical grade I varicocele, 36 (36.7%) had clinical grade II varicocele, and 59 (60.2%) had clinical grade III varicocele.

Prior to varicocelectomy, 45 of 101 (44.6%) men had TMSC >9M and would be candidates for NP, 20 (19.8%) had TMSC 5-9M and were candidates for IUI, and 36 (35.6%) men had TMSC <5M and would require ICSI/IVF for family planning. The median time to follow-up SA was 6.6 months (IQR 3.9-13.6 months).

Overall, median TMSC increased from 7.6M (IQR 2.6-21.4M) to 14.5 (IQR 2.8-33M) (mean difference 6.4M, 95% confidence interval 1.0-19.6M, p=0.02) after surgical repair (Table 1). Men with baseline TMSC <5M (IVF/ICSI) had increased median TMSC from 1.3M (IQR 0-2.9M) to 1.8M (IQR 0-7.5M) (mean difference 6.8M, 95% CI 0.4-13.2M, p=0.04). Men with baseline TMSC 5-9M (IUI) increased from a median of 6.9M (IQR 5.8-7.7M) to 12.0M (IQR 3.2-24.6M) (mean difference 15.6M, 95% CI 0.1-31.2M, p=0.05). Men with baseline TMSC >9M (NP) had no statistically significant improvement in TMSC following varicocelectomy (median 24.5M [IQR 13.9-63.8M] to 26.1M [IQR 17.6-47.3M], mean difference 2.0M [95% CI -6.8 to 10.7M], p=0.66). After surgical repair, among all patients, 35 (34.7%) patients had decreased TMSC on postoperative SA, and 11 (10.9%) men were “downgraded” from IUI to IVF (n=6), or from NP to IUI (n=4) or IVF (n=1) (Table 2).

**Table 1:** Changes in total motile sperm count (TMSC) after varicocele repair. IUI = intrauterine insemination, IVF = in vitro fertilization, NP = natural pregnancy. Continuous variables are represented as median (interquartile range).

Characteristic	Patients	Preoperative TMSC (Million)	Postoperative TMSC (Million)	p-value (<0.05)
IVF (<5 Million)	36	1.3 (0-2.9)	1.8 (0-7.5)	0.04
IUI (5-9 Million)	20	6.9 (5.8-7.7)	12.0 (3.2-24.6)	0.05
NP (>9 Million)	45	24.5 (13.9-63.8)	26.1 (17.6-47.3)	0.66
All Men	101	7.6 (2.6-21.4)	14.5 (2.8-33)	0.02

**Table 2:** Changes in assisted reproductive technology candidacy after varicocelectomy. IUI = intrauterine insemination, IVF = in vitro fertilization, NP = natural pregnancy, TMSC = total motile sperm count

		Post-varicocelectomy TMSC		
Characteristic	Patients	IVF, n (%)	IUI, n (%)	NC, n (%)
IVF (<5 Million)	36	23 (63.9)	5 (13.9)	8 (22.2)
IUI (5-9 Million)	20	6 (30.0)	3 (15.0)	11 (55.0)
NP (>9 Million)	45	1 (2.2)	4 (8.9)	40 (88.9)

There were no differences in baseline demographics including age, race, and ethnicity, or clinical characteristics including genitourinary-related medical comorbidities, testicular volume, preoperative hormonal profiles, or varicocele grade between men with increased or equal TMSC postoperatively compared to men with decreased TMSC following varicocelectomy. Demographic and clinical data are shown in Table 3. However, a comparison of men who experienced clinical downgrading after varicocelectomy showed they had significantly larger left testicular volume compared to men who did not experience clinical downgrading (20 mL [IQR 18-20] vs 16 mL [IQR 14-18], p=0.01, Table 4).

**Table 3:** Demographics and clinical characteristics between patients with and without total motile sperm count (TMSC) decrease on post-varicocelectomy semen analysis. BMI = body mass index, FSH = follicle stimulating hormone, LH = luteinizing hormone. Continuous variables are represented as median (interquartile range). Categorical data are represented as n (%).

	Equal/Increased TMSC N = 66 (%)	Decreased TMSC N = 35 (%)	P-value (p<0.05)
Age (years)	33 (30-36)	33 (31-35)	0.55
BMI	26.1 (24.3-28.0)	25.1 (23.0-27.0)	0.11
Race/Ethnicity			0.54
White	42 (71.2)	29 (85.3)	
Asian	5 (8.5)	1 (2.9)	
Black/African American	8 (13.6)	3 (8.8)	
Other/Unknown	4 (6.8)	1 (2.9)	
Medical Comorbidities			
Cryptorchidism	2 (3.0)	0 (0.0)	0.54
Hypertension	4 (6.1)	1 (2.9)	0.66
Chronic Kidney Disease	2 (3.0)	1 (2.9)	>0.99
Cancer History	1 (1.5)	1 (2.9)	>0.99
Testicular Volume (mL)			
Left Testis	17 (14-18)	18 (16-20)	0.21
Right Testis	18 (16-20)	18 (15-20)	0.33
Preoperative Hormone Level			
Testosterone (ng/dL)	408 (306-500)	378 (329-443)	0.96
LH (mIU/mL)	4.1 (2.9-6.2)	4.1 (3.2-5.2)	0.33
FSH (mIU/mL)	5.7 (3.9-9.5)	6.9 (5.1-8.7)	0.45
Clinical Grade			
Left Varicocele			0.10
Grade I	1 (1.6)	3 (8.6)	
Grade II	26 (41.3)	9 (25.7)	
Grade III	36 (57.1)	23 (65.7)	
Right Varicocele			0.89
Grade I	9 (26.5)	7 (33.3)	
Grade II	23 (67.7)	13 (61.9)	
Grade III	2 (5.9)	1 (4.8)	
Laterality of Repair			0.67
Unilateral (Left)	31 (47.0)	14 (41.2)	
Bilateral	35 (53.0)	21 (58.8)	

**Table 4:** Demographics and clinical characteristics between patients with and without clinical downgrading on post-varicocelectomy semen analysis. BMI = body mass index, FSH = follicle stimulating hormone, LH = luteinizing hormone. Continuous variables are represented as median (interquartile range). Categorical data are represented as n (%).

	No Clinical Downgrade N = 90 (%)	Clinical Downgrade N = 11 (%)	P-value (p<0.05)
Age (years)	33 (30-36)	33 (31-35)	0.79
BMI	25.8 (24.0-27.5)	26.0 (22.3-27.1)	0.94
Race/Ethnicity			0.65
White	69 (76.7)	10 (90.9)	
Asian	6 (6.7)	0 (0.0)	
Black/African American	10 (11.1)	1 (9.1)	
Other/Unknown	5 (5.6)	0 (0.0)	
Medical Comorbidities			
Hypertension	4 (4.4)	1 (9.1)	0.45
Cancer History	1 (1.1)	1 (9.1)	0.21
Testicular Volume (mL)			
Left Testis	16 (14-18)	20 (18-20)	0.01
Right Testis	18 (16-20)	18 (18-20)	0.60
Preoperative Hormone Level			
Testosterone (ng/dL)	393 (309-483)	397 (361-478)	0.64
LH (mIU/mL)	4.0 (2.8-5.6)	4.4 (3.8-6.2)	0.89
FSH (mIU/mL)	6.6 (4.1-9.4)	7.1 (5.1-10.4)	0.81
Clinical Grade			
Left Varicocele			0.84
Grade I	4 (4.6)	0 (0.0)	
Grade II	30 (34.5)	5 (45.5)	
Grade III	53 (60.9)	6 (54.5)	
Right Varicocele			0.46
Grade I	14 (29.2)	2 (28.6)	
Grade II	32 (66.7)	4 (57.1)	
Grade III	2 (4.2)	1 (14.3)	
Laterality of Repair			0.75
Unilateral (Left)	41 (45.6)	4 (36.4)	
Bilateral	49 (54.4)	7 (63.6)	

On logistic regression analysis, larger left testicle size was associated with clinical “downgrading” after varicocelectomy (OR 1.4, 95% CI 1.1-1.8, p=0.02) (Table 5). Also, preoperative clinical grade III varicocele was associated with decreased sperm motility on postoperative SA (OR 4.1, 95% CI 1.7-10.0, p=0.002).

**Table 5:** Logistic regression analysis of demographic and clinical characteristics associated with decreased semen parameters following varicocelectomy. BMI = body mass index, CI = confidence interval, FSH = follicle stimulating hormone, LH = luteinizing hormone, TMSC = total motile sperm count

	Concentration OR (95% CI)	p-value <0.05	Motility OR (95% CI)	p-value <0.05	TMSC OR (95% CI)	p-value <0.05	“Downgrade” OR (95% CI)	p-value <0.05
Age at surgery (years)	1.0 (0.9-1.1)	0.94	1.0 (0.9-1.1)	0.80	1.0 (0.9-1.1)	0.54	1.0 (0.9-1.1)	0.79
BMI	0.9 (0.8-1.1)	0.29	1.0 (0.9-1.1)	0.65	0.9 (0.8-1.0)	0.11	1.0 (0.8-1.2)	0.94
Testicular Volume								
Left testis (mL)	1.1 (1.0-1.2)	0.25	1.0 (0.9-1.1)	0.64	1.1 (1.0-1.2)	0.21	1.4 (1.1-1.8)	0.02
Right testis (mL)	1.0 (0.9-1.1)	0.72	0.9 (0.8-1.0)	0.18	1.0 (0.9-1.1)	0.33	1.0 (0.9-1.2)	0.59
Preoperative labs								
FSH (mIU/mL)	1.0 (0.9-1.0)	0.49	1.0 (0.9-1.0)	0.33	1.0 (0.9-1.0)	0.45	1.0 (0.9-1.1)	0.81
LH (mIU/mL)	0.9 (0.8-1.1)	0.38	0.8 (0.6-1.0)	0.11	0.9 (0.8-1.1)	0.37	1.0 (0.9-1.2)	0.97
Testosterone (ng/dL)	1.0 (1.0-1.0)	0.95	1.0 (1.0-1.0)	0.40	1.0 (1.0-1.0)	0.96	1.0 (1.0-1.0)	0.63
Clinical Grade								
Grade I/II	Ref.	–	Ref.	–	Ref.	–	Ref.	–
Grade III	0.8 (0.4-1.9)	0.66	4.1 (1.7-10.0)	0.002	1.6 (0.7-3.7)	0.28	0.8 (0.2-3.0)	0.78

A total of 45/101 (44.6%) of men had multiple postoperative SA following varicocelectomy. Among this cohort, the median time to first postoperative SA was 3.9 months (IQR 3.1-6.0 months) and the median time to last postoperative SA was 12.2 months (IQR 7.1-25.4 months). Among patients who were candidates for IVF after their first postoperative SA, 11.8% (2/17) were “upgraded” to IUI (n=1) or NP (n=1) on the last postoperative SA. Among patients who were candidates for IUI after the first postoperative SA, 40.0% (n=2) were “downgraded” to IVF, 40.0% (n=2) were “upgraded” to NP, and 20.0% (n=1) remained candidates for IUI on last postoperative SA. Lastly, among patients who were candidates for NP after the first postoperative SA, 8.7% (n=2) were “downgraded” to IVF on the last postoperative SA (Table 6).

**Table 6:** Changes in assisted reproductive technology candidacy between first postoperative SA to last postoperative SA (n=45). IUI = intrauterine insemination, IVF = in vitro fertilization, NP = natural pregnancy, SA = semen analysis, TMSC = total motile sperm count

		Last Post-varicocelectomy TMSC Category		
Characteristic	Patients	IVF, n (%)	IUI, n (%)	NC, n (%)
IVF (<5 Million)	17	15 (88.2)	1 (5.9)	1 (5.9)
IUI (5-9 Million)	5	2 (40.0)	1 (20.0)	2 (40.0)
NP (>9 Million)	23	2 (8.7)	0 (0.0)	21 (91.3)

## Discussion

Our study is the first to investigate predictive factors associated with decreased semen parameters and clinical “downgrading” of TMSC following varicocelectomy. We showed that a small, but significant, subset of men experience semen parameter deterioration after surgical repair. Men with larger left testicular volume were more likely to have clinical “downgrading” after varicocelectomy. Clinical grade III varicocele, diagnosed at the time of initial evaluation by an RU, was also associated with decreased sperm motility on postoperative SA. We also showed that 8.9% of men with multiple postoperative SA had “downgrading” from their initial postoperative SA, and similarly, 8.9% had clinical “upgrading” from their first postoperative SA to their last postoperative SA. This shows that variations in SA can occur in a bidirectional pattern and therefore should be considered in the context of our results. Lastly, men with baseline TMSC >9M did not experience significant improvement in postoperative SA and may be counseled against varicocelectomy for concerns of infertility.

Overall, our median follow-up from surgery to patients’ last postoperative SA was 6.6 months, which provides adequate time for semen parameters to improve or change after varicocelectomy. Prior studies show that among patients with preoperative TMSC <5M (IVF) and preoperative TMSC 5-9M (IUI), postoperative TMSC does not improve past the third and sixth month, respectively [16]. Similarly, in a study of 170 men, 118 of whom experienced a 50% or greater improvement in their TMSC after varicocelectomy, only five (2.9%) experienced this improvement >6 months after their procedure [17].

We grouped men into three classifications according to TMSC. Men with >9M were considered candidates for NP, 5-9M were candidates for IUI, and <5M were candidates for IVF. This classification has been previously described by Samplaski et al. to study the clinical “upgrading” of TMSC following varicocelectomy. In this study, the authors evaluated 373 men after surgical repair and found 112/373 (30.0%) of men had clinical “upgrading” [10]. Among our cohort, we found 24 of 101 (23.8%) of men had clinical “upgrading.” Our rate of clinical “downgrading” (10.6%), however, is similar to the reported rate also found by Samplaski et al. (12.6%). Although the authors compared their rate of clinical “downgrading: to a matched-control cohort to confirm varicocelectomy did not increase the risk of clinical “downgrading,” they did not examine specific characteristics or preoperative factors associated with “downgrading,” which was the impetus for the current study.

We found that preoperative clinical grade III varicocele was associated with decreased sperm motility, but not TMSC, following varicocelectomy. Prior studies have focused on varicocele grade and its association with baseline semen parameters, as well as its utility as a prognostic indicator of success for surgical repair. However, the results are conflicting. Steckel et al. showed that higher varicocele grade was associated with significantly improved sperm motility on postoperative SA, with grade III patients experiencing the most robust clinical response (30 ± 4% vs 41 ± 4%, p<0.01) [18]. Likewise, Samplaski et al. published a nomogram for expected postoperative semen changes which also identified clinical varicocele grade as a significant factor associated with increased sperm concentration, ejaculate volume, sperm motility, morphology, and TMSC after surgery [12]. In contrast, Ghayda et al. showed that clinical grades I and II experienced increased sperm motility and sperm concentration after varicocelectomy, however, clinical grade III experienced no significant improvements in any semen parameters included in their analyses [19]. One explanation for this somewhat paradoxical finding could be the role of irreversible deterioration of testicular function in men with clinical grade III varicocele. The current study found a significant association between grade III varicoceles and poor post-operative sperm motility, consistent with this hypothesis. However, poor motility was not associated with overall impaired TMSC or “downgrading,” so the clinical significance of this finding is unclear.

We found that larger left testicular size was associated with “downgrading” after varicocelectomy. One possible explanation is that varicocele-related infertility results in a degree of testicular atrophy, specifically on the left side given the predisposition of varicoceles to impact the left testis [20]. As such, a larger or normal left testis prior to repair may indicate that abnormal semen parameters are not indeed varicocele-related. Therefore, surgical repair of the varicocele may not correct the underlying pathology or etiology of impaired spermatogenesis in that individual. However, further studies are needed to validate these findings.

Based on our results, couples should be informed that a proportion of men undergoing varicocele repair will experience “downgrading” in semen parameters. Prior studies show that varicocelectomy can improve semen parameters and therefore improve chances of natural conception [21]. Schlegel et al. found varicocelectomy to be cost-effective, estimating the cost per live birth after varicocele repair to be $26,268, compared with $89,091 after intracytoplasmic sperm injection [22]. However, for men who experience clinical “downgrading,” the financial implications are reversed. Couples who were previously candidates for NP will now require more invasive approaches with substantial morbidity to the female partner. They will also be pursuing these therapies in a delayed fashion, many months after they initially sought treatment. These couples may incur the added costs of IUI or IVF, which are substantial. A 2014 analysis estimated the median out-of-pocket expense for fertility treatment to be $5,338, with the highest cost incurred for IVF ($19,234). Additionally, live birth rates of 50% per intended egg retrieval cycle for IVF require couples to often undergo multiple treatments, which can cost $6,955 for each additional cycle [23,24]. These potential clinical and financial risks, coupled with the low, but potential risk of testicular atrophy (<1%), varicocele recurrence (1-3%), and postoperative pain (8-10%), are important for patient counseling prior to surgical intervention [25,26].

Our study must be considered in the context of certain limitations. First, the retrospective nature of this study relied on previously collected data in the electronic database, which may introduce bias due to incomplete reporting or incorrect coding. Second, our data was restricted to men with follow-up SA results in our healthcare system beyond the immediate postoperative period. Third, while we did have a robust follow-up of approximately seven months, which is beyond the time that most improvement is seen after varicocelectomy, we could not determine whether improvements or declines in semen parameters were sustained in the longer term. This is a limitation of most varicocelectomy studies, since most couples have either achieved pregnancy or moved on to ARTs after this period of time, and these couples often do not return for repeat evaluation [27]. This may introduce selection bias by way of including only those men who were motivated to undergo follow-up SA and excluding men who had follow-up SA at outside institutions or those who did not undergo follow-up SA. While all semen samples were analyzed according to WHO 2010 Guidelines, inter-lab variability cannot be adequately controlled between samples. Lastly, we included only one preoperative and one postoperative SA in our primary analysis, despite known variability in semen parameters over time. This could over- or under-estimate the proportion of men who experienced downgrading after surgery. We attempted to address this limitation with a sub-group analysis of patients with multiple postoperative SAs available for review.

## Conclusions

Approximately one in 10 men may experience clinical “downgrading” of TMSC following varicocelectomy. Larger left testis size was associated with clinical downgrading, whereas clinical grade III varicoceles were associated with lower post-treatment sperm motility, but not a decline in TMSC. Our analysis provides valuable information for preoperative counseling for both patients and clinicians and can help to identify men who will not benefit from surgical intervention. Further studies are required to validate our findings and to better determine predictors of negative outcomes following varicocelectomy.
